# Supplementary material for: Viscoelasticity in simple indentation-cycle experiments: a computational study
Source: Sci Rep. 2020 Aug 6;10:13302. doi: 10.1038/s41598-020-70361-y (PMC7413555; doi:10.1038/s41598-020-70361-y)
Supplement: Supplementary file 1 — Supplementary Information 1. [file 41598_2020_70361_MOESM1_ESM.pdf]

Electronic Supplementary Information

Viscoelasticity in simple indentation-cycle experiments: a computational study

Yu.M. Efremov[a*], S.L. Kotova[a,b], P.S. Timashev[a,b,c,d]

[a] Institute for Regenerative Medicine, Sechenov University, 8 Trubetskaya St., Moscow, 119991, Russia

[b] N.N. Semenov Institute of Chemical Physics, 4 Kosygin St., Moscow, 119991, Russia

[c] Institute of Photon Technologies of Federal Scientific Research Centre "Crystallography and Photonics" of Russian Academy of Sciences, Pionerskaya 2, Troitsk, Moscow, 108840, Russia

[d] Chemistry Department, Lomonosov Moscow State University, Leninskiye Gory 1-3, Moscow 119991, Russia

*Corresponding author: Yuri M. Efremov (yu.efremov@gmail.com)

Appendix A.

Analytical solutions of the Ting's equations for selected viscoelastic models, probe geometries and indentation histories.

For the acquisition of analytical solutions, the equations were solved symbolically with SageMath [1] and Wolfram Alpha integral calculator [2]. We will reproduce the relaxation functions here and provide analytical solutions for the Ting's equations describing a common indentation experiment with the triangular or sinusoidal displacement. The solutions for the approach (tip deepens into the sample, contact area increasing) and retraction (contact area decreasing) curves will be provided separately. The solutions for the approach curve (Lee-Radok's solution) could also be extended for the case of the retraction curves, we did it for some situations where the complete Ting's solution was not obtained. The Lee-Radok's and Ting's solution match for the cylindrical probe since the contact area is constant or has a zero value.

The **spring element**, $\sigma(t)=k\varepsilon(t)$. The relaxation function is constant in time (*E(t)=E*), and the Ting's equation solution corresponds to the well-known Hertzian solution of the form:

$$F\left(\delta\left(t\right)\right)=C_{geom}E\delta^{n}. \quad \text{(A1)}$$

The n=1, 1.5, 2 for the cylindrical, spherical and conical probe, respectively, the geometrical coefficients are: $C_{cylinder} = 2R_c/\left(1-\nu^2\right)$, $C_{sphere}=4\sqrt{R}/3/\left(1-\nu^2\right)$, $C_{cone}=2\left(tan\,\alpha\right)/\pi/\left(1-\nu^2\right)$.

The **dashpot element**, $\sigma(t) = \eta d\varepsilon(t)/dt$ (η is the viscoelastic coefficient or viscosity) according to the behavior of an ideal Newton liquid. The relaxation function is $E(t) = \eta\delta_D(t)$, where $\delta_D(t)$ is the Dirac delta function. The common analytical solution of the Ting's equation for all the geometries is:

$$F_{appr}\left(\delta(t)\right) = C_{geom} n\eta\delta^{n-1}(t)\dot{\delta}(t); \quad \text{(A2)}$$

$$F_{retr}\left(\delta(t)\right) = 0; \quad \text{(A3)}$$

$$t_1(t, t > t_\text{m}) = 0. \quad \text{(A4)}$$

As expected for a viscous material, the force drops to zero then the cantilever goes up (retracts).

The **Kelvin-Voight element**, a combination of a spring and a dashpot in parallel, has the following relaxation function: $E(t) = E_\infty + \eta\delta_D(t)$. We will split the solutions for the approach F_{ap} and retraction F_{retr} curves. $t_1(t)$ function can be found from Eq. (3), which leads to the condition:

$$\delta(t_1, t > t_m) = \delta(t) + \frac{\eta}{E}\dot{\delta}(t_1); \quad \text{(A5)}$$

which for the triangular ramp leads to:

$$t_1(t) = 2t_m - t - \frac{\eta}{E}; \quad \text{(A6)}$$

and for the sinusoidal ramp:

$$t_1(t > t_m) = \frac{1}{\omega}\arcsin\left(\sqrt{1+\left(\frac{\eta\omega}{E}\right)^2}\sin\left(\omega t + \tan^{-1}\left(\frac{\eta\omega}{E}\right)\right)\right). \quad \text{(A7)}$$

The common solution for all the geometries for the triangular (ramp) and sinusoidal (sin) indentation histories are, respectively:

$$F_{appr,ramp}\left(\delta(t)\right) = C_{geom}(vt_1)^{n-1}\left(Evt_1 + n\eta v\right); \quad \text{(A8)}$$

$$F_{retr,ramp}\left(\delta(t)\right) = C_{geom}\left(vt_1\right)^n E = C_{geom}\left(v(2t_m - t - \frac{\eta}{E})\right)^n E; \quad \text{(A9)}$$

$$F_{appr,\sin}\left(\delta(t)\right) = C_{geom}(A\sin(\omega t_1))^{n-1}\left(EA\sin(\omega t_1) + n\eta A\omega\cos(\omega t_1)\delta_{K[t_1,t]}\right); \quad \text{(A10)}$$

$$F_{retr,\sin}\left(\delta(t)\right) = C_{geom}\left(A\sin(\omega t_1)\right)^n E; \quad \text{(A11)}$$

solution for the approach curve was also obtained before in [3].

For the **Maxwell element**, the relaxation function is: $E(t)=E_0 e^{-\frac{t}{\tau}}$. The $t_1(t)$ function for the triangular displacement is:

$$t_1 = t + \tau \ln(2e^{-\frac{t-t_m}{\tau}} - 1); \qquad \text{(A12)}$$

The solution for $t_1(t)$ in the case of the sinusoidal load could not be isolated, but it can be numerically found from the following relation:

$$e^{\frac{t_1-t}{\tau}}(\tau\omega\sin(t_1\omega)+\cos(t_1\omega)) = \tau\omega\sin(t\omega)+\cos(t\omega). \qquad \text{(A13)}$$

For the approach curve, the solutions for the triangular displacement and different probe geometries are:

$$F_{appr,cyl,ramp}\left(\delta(t)\right) = C_{geom}E_0 v\tau(1-e^{-\frac{t}{\tau}}); \qquad \text{(A14)}$$

$$F_{appr,sphere,ramp}\left(\delta(t)\right) = C_{geom}E_0\frac{3}{2}(v\tau)^{\frac{3}{2}}\left(\sqrt{\frac{t}{\tau}} - \sqrt{\frac{\pi}{4}}erfi\left(\sqrt{\frac{t}{\tau}}\right)e^{-\frac{t}{\tau}}\right); \qquad \text{(A15)}$$

$$F_{appr,cone,ramp}\left(\delta(t)\right) = C_{geom}E_0 2(v\tau)^2(\frac{t}{\tau}-1+e^{-\frac{t}{\tau}}); \qquad \text{(A16)}$$

$$F_{retr,cyl,ramp}\left(\delta(t)\right) = C_{geom}E_0 v\tau(2e^{\frac{-t+t_m}{\tau}} - e^{-\frac{t}{\tau}} - 1); \qquad \text{(A17)}$$

$$F_{retr,sphere,ramp}\left(\delta(t)\right) = C_{geom}E_0 3(v\tau)^{\frac{3}{2}} i\left(-\sqrt{\frac{\pi}{16}}e^{-\frac{t}{\tau}}erfi\left(\sqrt{\ln(2e^{\frac{t_m-t}{\tau}}-1)+\frac{t}{\tau}}\right) + \left(e^{\frac{t_m-t}{\tau}} - \frac{1}{2}\right)\sqrt{\ln(2e^{\frac{t_m-t}{\tau}}-1)+\frac{t}{\tau}}\right)$$

$$; \qquad \text{(A18)}$$

$$F_{retr,cone,ramp}\left(\delta(t)\right) = C_{geom}E_0 4(v\tau)^2(\left(e^{\frac{t_m-t}{\tau}} - \frac{1}{2}\right)\ln\left(2e^{\frac{t_m-t}{\tau}}-1\right) + \frac{t-\tau}{\tau}e^{\frac{t_m-t}{\tau}} + \frac{1}{2}e^{-\frac{t}{\tau}} - \frac{t}{2\tau} + \frac{1}{2})$$

$$; \qquad \text{(A19)}$$

where *erfi()* is the imaginary Gauss error function. For the sinusoidal displacement, there is no closed-form analytical solution for the case of the spherical probe:

$$F_{appr,cyl,\sin}\left(\delta(t)\right) = C_{geom}E_0 A\omega\tau(\frac{\tau\omega\sin(t\omega)+\cos(t\omega)-e^{-\frac{t}{\tau}}}{(t\omega)^2+1}); \qquad \text{(A20)}$$

$$F_{appr,sphere,\sin}\left(\delta(t)\right)=C_{geom}A\omega\frac{3}{2}\int_0^t e^{-\frac{(t-x)}{\tau}}\sqrt{\sin(\omega x)}\cos(\omega x)dx; \quad \text{(A21)}$$

$$F_{appr,cone,\sin}\left(t\right)=C_{geom}E_0(A\omega\tau)^2\left(\frac{\frac{2\sin(t\omega)\cos(t\omega)}{\tau\omega}-4\cos(t\omega)^2+2e^{-\frac{t}{\tau}}+2}{4(t\omega)^2+1}\right); \quad \text{(A22)}$$

$$F_{retr,cyl,\sin}\left(\delta(t)\right)=C_{geom}E_0A\omega\tau\left(\frac{e^{\frac{t_1-t}{\tau}}\left(\tau\omega\sin(t_1\omega)+\cos(t_1\omega)\right)-e^{-\frac{t}{\tau}}}{(t\omega)^2+1}\right); \quad \text{(A23)}$$

$$F_{retr,sphere,\sin}\left(\delta(t)\right)=C_{geom}A\omega\frac{3}{2}\int_0^{t_1} e^{-\frac{(t-x)}{\tau}}\sqrt{\sin(\omega x)}\cos(\omega x)dx; \quad \text{(A24)}$$

$$F_{ret,cone,\sin}\left(t\right)=C_{geom}E_0(A\omega\tau)^2e^{-\frac{t}{\tau}}\left(\frac{e^{\frac{t_1}{\tau}}\left(\frac{\sin(2t_1\omega)}{\tau\omega}-2\cos(2t_1\omega)\right)+2}{4(t\omega)^2+1}\right). \quad \text{(A25)}$$

By comparing Eqs. A12-14 and A23, it follows that for the cylindrical indenter the solutions for the approach and retraction curves match, as stated above. It is true for other considered viscoelastic models as well.

The relaxation function of the **Standard linear solid** model is $E(t)=(E_0-E_{\inf})e^{-\frac{t}{\tau}}+E_{\inf}$. The $t_1(t)$ function for the triangular ramp is:

$$t_1=2t_m-t+\tau\left(\left(2e^{-\frac{t-t_m}{\tau}}-1\right)\left(\frac{E_0}{E_{\inf}}-1\right)-W\left(\left(\frac{E_0}{E_{\inf}}-1\right)e^{\left(2e^{-\frac{t-t_m}{\tau}}-1\right)\left(\frac{E_0}{E_{\inf}}-1\right)+\frac{2(t_m-t)}{\tau}}\right)\right); \quad \text{(A26)}$$

where W is the Lambert W function. For the sinusoidal displacement, the $t_1(t)$ function can be numerically found from the following relation:

$$e^{\frac{t_1-t}{\tau}}(\tau\omega\sin(t_1\omega)+\cos(t_1\omega))(E_0-E_{\inf})-E_{\inf}(\tau\omega+(\tau\omega)^{-1})\sin(t_1\omega)=(E_0-E_{\inf})\cos(t\omega)+(E_0\tau\omega+E_{\inf}(\tau\omega)^{-1})\sin(t\omega)$$

. (A27)

The solutions for the approach curves are:

$$F_{appr,cyl,ramp}\left(\delta(t)\right)=C_{geom}v(-(E_0-E_{\inf})\tau e^{-\frac{t}{\tau}}+(E_0-E_{\inf})\tau+E_{\inf}t); \quad \text{(A28)}$$

$$F_{appr,sphere,ramp}\left(\delta(t)\right)=C_{geom}v^{\frac{3}{2}}\left(-\frac{3}{4}(E_0-E_{inf})\sqrt{-\pi\tau^3}erf\left(\sqrt{-\frac{t}{\tau}}\right)e^{-\frac{t}{\tau}}+\frac{3}{2}(E_0-E_{inf})\tau\sqrt{t}+E_{inf}t^{\frac{3}{2}}\right)$$

; (A29)

$$F_{appr,cone,ramp}\left(\delta(t)\right)=C_{geom}v^2(2\tau^2(E_0-E_{\inf})(e^{-\frac{t}{\tau}}+\frac{t}{\tau}-1)+E_{\inf}t^2); \quad \text{(A30)}$$

For the retraction curves, the analytical solutions for the spherical and conical probes are too long and complex, so here only the solution for the cylindrical probe is presented:

$$F_{retr,cyl,ramp}\left(\delta(t)\right)=C_{geom}v(\tau(E_0-E_{\inf})(2e^{\frac{-t+t_m}{\tau}}-e^{-\frac{t}{\tau}}-1)-E_{\inf}(t-2t_m)). \quad \text{(A31)}$$

The solutions for the sinusoidal displacement were found except for the case of sphere-retraction, but are not presented here due to complexity.

For a single springpot element (Power-law rheology model), we will use the Young's relaxation function in the form of $E(t)=E_{\alpha 1}t^{-\alpha}$. The analytical solutions for the Ting's equations for triangular indentation were obtained previously by Bruckner et al. [4] and also used by Garcia et al. with some modifications [5]. The $t_1(t)$ function for the triangular ramp is:

$$t_1(t)=t-2^{\frac{1}{1-\alpha}}(t-t_m); \quad \text{(A32)}$$

the solution was not acquired for the sinusoidal ramp.

The solutions for the triangular ramp are as follows:

$$F_{appr,cyl,ramp}\left(\delta(t)\right)=C_{geom}v\frac{E_{\alpha 1}}{1-\alpha}t^{1-\alpha}; \quad \text{(A33)}$$

$$F_{appr,sphere,ramp}\left(\delta(t)\right)=C_{geom}v^{\frac{3}{2}}\frac{3\sqrt{\pi}}{4}\frac{E_{\alpha 1}\Gamma(1-\alpha)}{\Gamma(\frac{5}{2}-\alpha)}t^{\frac{3}{2}-\alpha}; \quad \text{(A34)}$$

$$F_{appr,cone,ramp}\left(\delta(t)\right)=C_{geom}v^2\frac{2E_{\alpha 1}}{\alpha^2-3\alpha+2}t^{2-\alpha}; \quad \text{(A35)}$$

$$F_{retr,cyl,ramp}\left(\delta(t)\right)=C_{geom}v\frac{E_{\alpha 1}}{1-\alpha}(t^{1-\alpha}-2(t-t_m)^{1-\alpha}); \quad \text{(A36)}$$

The solutions for the retraction curves for the spherical and conical geometries are too long and not presented here. For the sinusoidal ramp, only the following solutions were obtained for the approach curves:

$$F_{appr,cyl,\sin}\left(\delta(t)\right)=C_{geom}A\omega\frac{t^{1-\alpha}}{1-\alpha}\mathbb{F}\left[1,(1-\frac{\alpha}{2},\frac{3}{2}-\frac{\alpha}{2}),-\frac{(t\omega)^2}{4}\right]; \quad \text{(A37)}$$

$$F_{appr,cone,\sin}\left(\delta(t)\right)=C_{geom}\left(A\omega\right)^2\frac{2t^{2-\alpha}}{\alpha^2-3\alpha+2}\mathbb{F}\left[1,(\frac{3}{2}-\frac{\alpha}{2},2-\frac{\alpha}{2}),-(t\omega)^2\right]; \quad \text{(A38)}$$

where $\mathbb{F}(a,b,z)$ is the generalized hypergeometric function. The analytical solutions could be obtained for some other viscoelastic functions and particular sets of indentation histories and probe geometries, but it is beyond the tasks of the current study.

References

[1] The Sage Developers, SageMath, the Sage Mathematics Software System (Version 8.7), (2019). https://www.sagemath.org.

[2] Wolfram Research, WolframAlpha integral calculator, (2019). https://www.wolframalpha.com/calculators/integral-calculator.

[3] P.D. Garcia, C.R. Guerrero, R. Garcia, Time-resolved nanomechanics of a single cell under the depolymerization of the cytoskeleton, Nanoscale. 9 (2017) 12051–12059. https://doi.org/10.1039/C7NR03419A.

[4] B.R. Brückner, H. Nöding, A. Janshoff, Viscoelastic Properties of Confluent MDCK II Cells Obtained from Force Cycle Experiments, Biophys. J. 112 (2017) 724–735. https://doi.org/10.1016/j.bpj.2016.12.032.

[5] P.D. Garcia, C.R. Guerrero, R. Garcia, Nanorheology of living cells measured by AFM-based force–distance curves, Nanoscale. 12 (2020) 9133–9143. https://doi.org/10.1039/C9NR10316C.

[6] Y.M. Efremov, A.I. Shpichka, S.L. Kotova, P.S. Timashev, Viscoelastic mapping of cells based on fast force volume and PeakForce Tapping, Soft Matter. 15 (2019) 5455–5463. https://doi.org/10.1039/C9SM00711C.

[7] Y.M. Efremov, W.-H. Wang, S.D. Hardy, R.L. Geahlen, A. Raman, Measuring nanoscale viscoelastic parameters of cells directly from AFM force-displacement curves, Sci. Rep. 7 (2017) 1541. https://doi.org/10.1038/s41598-017-01784-3.

Supplementary figures

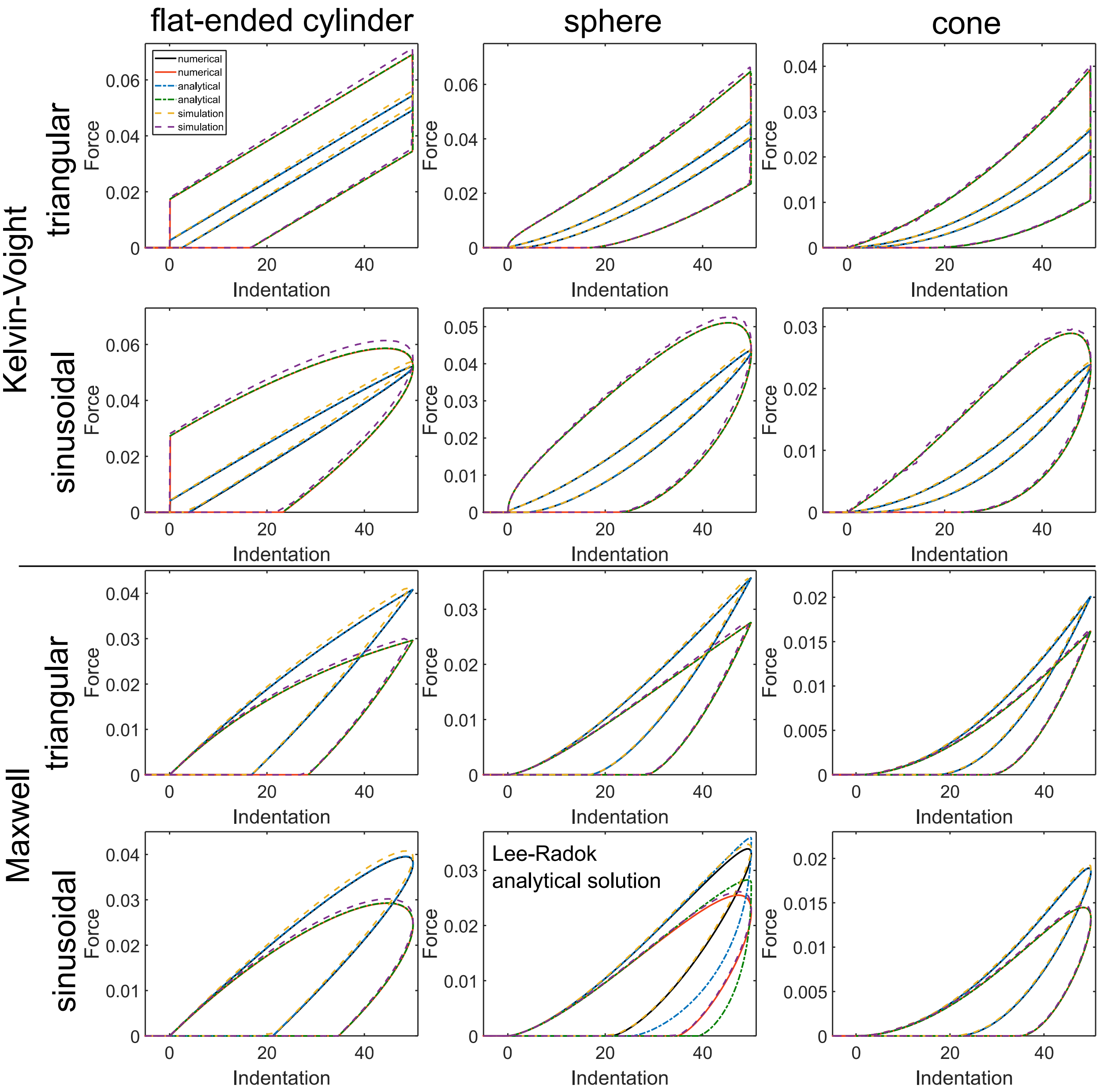

Figure S1. A comparison of numerical, analytical, and simulation solutions for the Kelvin-Voight and Maxwell models, different probe geometries (flat-ended cylinder, sphere, and cone) and indentation histories (triangular and sinusoidal probe displacement). The parameters of the Kelvin-Voight model: 1) E_∞=1000 Pa, η=10 Pa*s; 2) E_∞=1000 Pa, η=100 Pa*s. The parameters of the Maxwell model: 1) E_0=1000 Pa, τ=2 s; 2) E_0=1000 Pa, τ=0.8 s. The indentation speed for the triangular ramp was 50 nm/s, the frequency of the sinusoidal ramp was 0.25 Hz (total time was 2 s for both cases), the amplitude was 50 nm. There is no analytical Ting's solution for the Maxwell model-sphere-sinusoidal ramp case, the Lee-Radok's solution is presented.

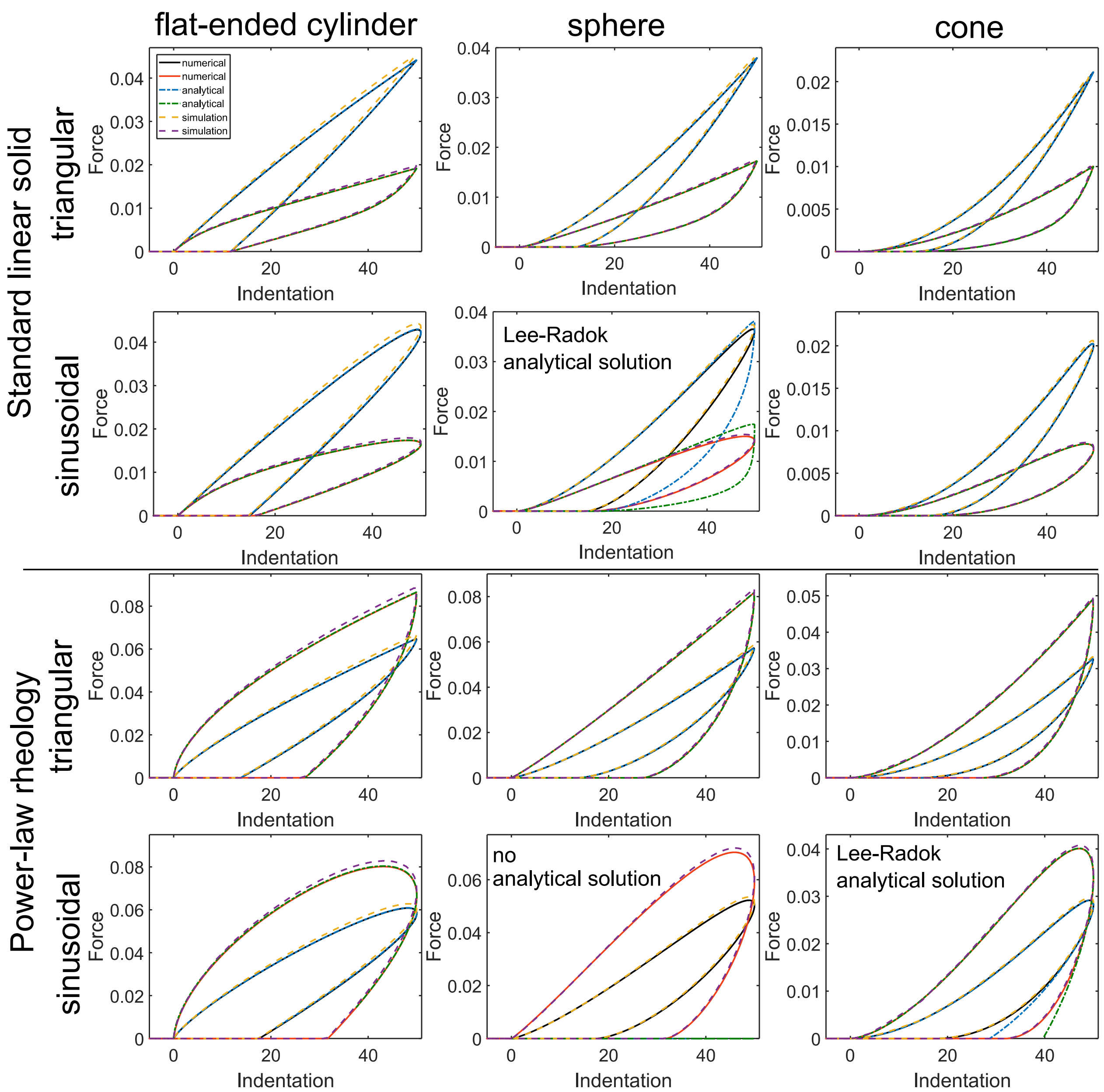

Figure S2. A comparison of numerical, analytical, and simulation solutions for the SLS and PLR models, different probe geometries (flat-ended cylinder, sphere, and cone) and indentation histories (triangular and sinusoidal probe displacement). The parameters of the SLS model: 1) E_0=1000 Pa, τ=2 s, E_∞=300 Pa; 2) E_0=1000 Pa, τ=0.5 s, E_∞=300 Pa. The parameters of the PLR model: 1) $E_{\alpha 1}$=1000 Pa, =0.2; 2) E_0=1000 Pa, α=0.4. The indentation speed for the triangular ramp was 50 nm/s, the frequency of the sinusoidal ramp was 0.25 Hz (total time was 2 s for both cases), the amplitude was 50 nm. There is no analytical Ting's solution for the SLS model-sphere-triangular ramp, PLR model-sphere-cone-sinusoidal ramp cases, the Lee-Radok's solution is presented where available.

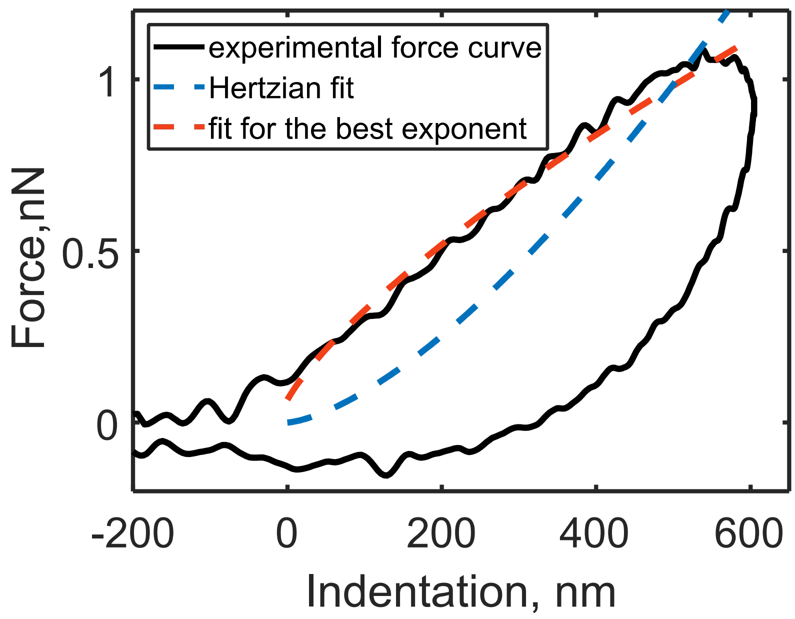

Figure S3. A force curve obtained with AFM indentation on a cell (NIH 3T3 fibroblast) at a high indentation rate. The force curve was obtained at the indentation rate of 660 Hz that corresponds to the indentation time of 0.0015 s, 140 nm diameter spherical (parabolical) probe, sinusoidal displacement. Due to a strong dissipation (the NHA=0.81), the Hertzian fit does not follow the curve closely, and the curve exponent (0.73) is twice lower than the Hertzian one (1.5). The experimental data are taken from [6].

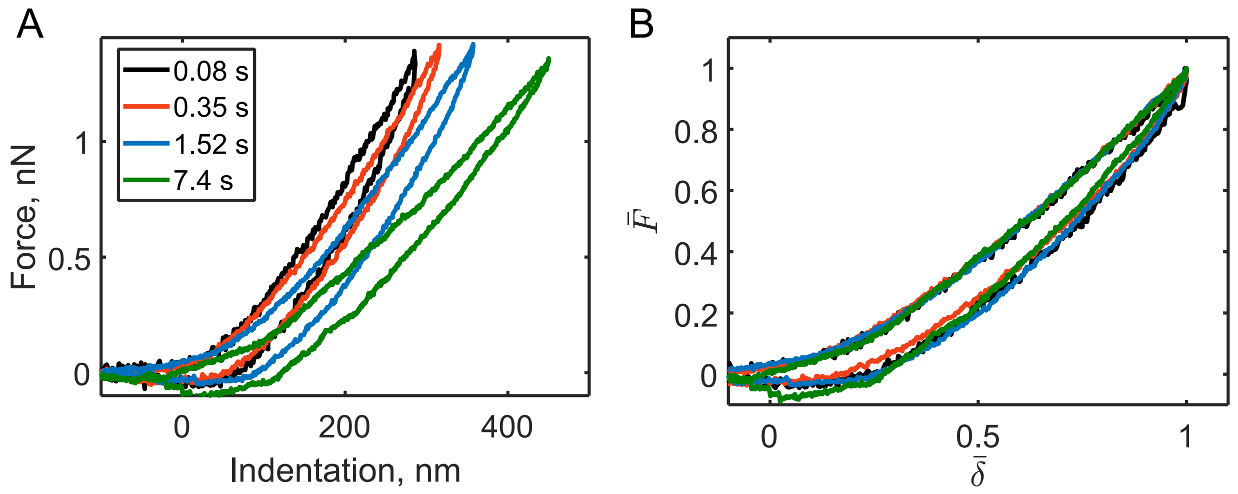

Figure S4. Force curves obtained with AFM indentation on cells (NIH 3T3 fibroblasts) can be described well with the power-law rheology model (single springpot). (A) The force curves obtained at different indentation times (showed in the legend) over three orders of magnitude, a 5 μm diameter spherical probe. (B) In the normalized coordinates, the curves match each other well since they all have very close values of the NHA and curve exponent. Moreover, these values are close to the values from the numerical prediction (NHA = 0.28 vs 0.26; curve exponent = 1.4 vs 1.4; for the experimental $\alpha \approx 0.1$). The experimental data are taken from [7].
